# Alterations in the Plasma Lipidome of Adult Women With Bipolar Disorder: A Mass Spectrometry-Based Lipidomics Research

**DOI:** 10.3389/fpsyt.2022.802710

**Published:** 2022-03-21

**Authors:** Lin Guo, Ting Zhang, Rui Li, Zhi-quan Cui, Jing Du, Jia-bin Yang, Fen Xue, Yi-Huan Chen, Qing-rong Tan, Zheng-wu Peng

**Affiliations:** ^1^Department of Psychiatry, Chang'an Hospital, Xi'an, China; ^2^Department of Psychiatry, Xijing Hospital, Air Force Medical University, Xi'an, China

**Keywords:** lipidomics, women, bipolar disorder, healthy controls, plasma

## Abstract

Lipidomics has become a pivotal tool in biomarker discovery for the diagnosis of psychiatric illnesses. However, the composition and quantitative analysis of peripheral lipids in female patients with bipolar disorder (BD) have been poorly addressed. In this study, plasma samples from 24 female patients with BD and 30 healthy controls (HCs) were analyzed by comprehensive lipid profiling and quantitative validation based on liquid chromatography–mass spectrometry. Clinical characteristics and a correlation between the level of lipid molecules and clinical symptoms were also observed. We found that the quantitative alterations in several lipid classes, including acylcarnitine, lysophosphatidylethanolamine, GM2, sphingomyelin, GD2, triglyceride, monogalactosyldiacylglycerol, phosphatidylinositol phosphate, phosphatidylinositol 4,5-bisphosphate, phosphatidylethanolamine, phosphatidylserine, and lysophosphatidylinositol, were remarkably upregulated or downregulated in patients with BD and were positively or negatively correlated with the severity of psychotic, affective, or mania symptoms. Meanwhile, the composition of different carbon chain lengths and degrees of fatty acid saturation for these lipid classes in BD were also different from those of HCs. Moreover, 55 lipid molecules with significant differences and correlations with the clinical parameters were observed. Finally, a plasma biomarker set comprising nine lipids was identified, and an area under the curve of 0.994 was obtained between patients with BD and the HCs. In conclusion, this study provides a further understanding of abnormal lipid metabolism in the plasma and suggests that specific lipid species can be used as complementary biomarkers for the diagnosis of BD in women.

## Introduction

Bipolar disorder (BD) is a severe and episodic mental illness characterized by mood alterations between euthymia, major depression, and mania (1). It affects approximately 3% of the general population (2), and its estimated lifetime prevalence ranges from 0.5 to 2.4% worldwide (3, 4). Moreover, current drug treatments are effective in only 40–60% of cases and cause severe side effects (5, 6). The association of BD with impaired quality of life and the high economic burden have been widely accepted (7, 8). Accurate early detection and intervention during the prodromal stages of BD may help avert the burden by decreasing symptom severity and preventing progression to full disorder status (9, 10). Notably, BD is frequently misdiagnosed as a major depressive disorder (MDD) because of overlapping symptomology, later onset of mania, and frequent depressive episodes (11). The incorrect treatment of BD with antidepressant monotherapy increases the risk of mania and the frequency of episodes, both of which can have detrimental effects on disease prognosis (12, 13). Therefore, the development of specific biomarkers to increase the diagnostic accuracy of BD is required.

Women are nearly twice as likely to develop MDD during their lifetime compared with men (14, 15). Although there is no sex difference in the incidence rate of BD, women are more likely to have precipitous changes between depression and hypomania/mania and episodes of both depressive and manic symptoms (16, 17); therefore, the misdiagnosis of BD in women is more common than in men. Notably, women with BD are at an increased risk of having a serious episode of illness in relation to pregnancy and childbirth (18, 19), and BD is considered the sixth leading cause of disability among women of reproductive age (4). Furthermore, the objective biomarkers for diagnosis have not been clearly identified, and specific medications for the treatment of BD in women are clinically unsatisfactory. Therefore, clarifying the pathological features of BD in women may provide a basis for the development of new diagnostic methods and treatment strategies.

Lipids play an important role in regulating and maintaining neuronal development and function (20–22), and their involvement in modulating synaptic physiology, axonal membranes, and energy metabolism in the brain has been largely demonstrated (23–25). Recently, lipidomic analysis based on liquid chromatography (LC)–mass spectrometry (MS) has allowed for the comprehensive identification of hundreds to thousands of lipid molecular species in tissues and plasma (26, 27), and lipidomics has become established as a pivotal tool for biomarker discovery for the diagnosis of psychiatric diseases (28). Studies have observed the composition of peripheral fatty acid and phospholipid classes in BD (29–31), and a MS-based serum lipidomics study also identified the serum lipid profiles between BD and healthy control (HC) groups (32); however, there is still a need for quantitative analysis of the changes in lipid species. Currently, there is only an indirect and limited understanding of peripheral lipid characteristics in women with BD.

Considering the above-mentioned information, this study sought to determine the differences in lipidomic composition of plasma samples from female patients with BD and the HCs and to analyze the correlation between different lipid profiles and symptoms. These data provide insights into lipidomic alterations in female patients with BD and may guide further studies on the diagnosis of BD.

## Methods

### Subjects and Plasma Sampling

The study protocol was approved by the Chinese Clinical Trial Ethics Committee (approval no. ChiECRCT20200090) and was registered with the Chinese Clinical Trial Registry (registration no. ChiCTR2000032118). This study was performed in accordance with the Declaration of Helsinki. All participants volunteered to participate in this study and provided written informed consent. Twenty-four female patients (22–58 years of age) who met the criteria for BD of the fifth edition of the *Diagnostic and Statistical Manual of Mental Disorders* (DSM-5) were recruited from the Department of Psychiatry of Chang'an Hospital, Xi'an, China, along with 30 healthy females (22–53 years of age). All participants underwent physical examination. The Mini-International Neuropsychiatric Interview was used to screen for preexisting psychiatric disorders.

The Hamilton Depression Rating Scale (HAMD), Hamilton Anxiety Scale (HAMA), Positive and Negative Syndrome Scale (PANSS), and Bech–Rafaelsen Mania Rating Scale (BRMS) tests were independently administered by two psychiatrists who were blinded to the clinical status of the participants and had attended a training session on how to administer the tests before the start of the study. The exclusion criteria were diseases of the digestive system; obesity, which was defined as a body mass index (BMI) ≥ 28.0; hypertension; participants with a severely imbalanced diet, such as high-fat diet preferences; pregnancy, lactation, or menstrual period; and presence of other mental disorders according to the DSM-5 criteria. The same exclusion criteria used for the BD group were also applied to the HC group.

Blood samples were collected between 8 and 10 a.m. from all individuals under fasting conditions. The blood was collected in anticoagulant tubes and centrifuged at 1,600 rpm for 15 min. The obtained plasma was aliquoted into sterile cryopreservation tubes and stored in liquid nitrogen until further analysis.

### Quantitative Lipidomics

Experiments and data analysis were performed as previously described (33, 34) and were supported by Shanghai Applied Protein Technology Co., Ltd. For the sample preparation, plasma (100 μl) was accurately measured and spiked with appropriate amounts of internal lipid standards (SPLASH^®^ LIPIDOMIX^®^ Mass Spec Standard, methanol solution, AVANTI, 330707-1EA, Merck) and homogenized with 200 μl water and 240 μl methanol. Thereafter, 800 μl methyl tert-butyl ether was added, and the samples were subjected to ultrasonication for 20 min at 4°C and allowed to rest for 30 min at room temperature. The solution was then centrifuged at 10°C for 15 min (14,000 × *g*), and the supernatant was separated for analysis. For the LC-MS/MS method, the samples were separated using a UHPLC Nexera LC-30A ultra-high-performance LC system with a C18 column (ACQUITY UPLC CSH C18, 1.7 μm, 2.1 × 100 mm, Waters). The column temperature was 45°C, and the flow rate was 300 μl/min. The lipid extracts were redissolved in 200 μl of 90% isopropanol/acetonitrile and centrifuged at 14,000 × *g* for 15 min, and 3 μl of the sample was injected. Solvent A was acetonitrile–water (6:4, v/v) with 0.1% formic acid and 0.1 mM ammonium formate. Solvent B was acetonitrile–isopropanol (1:9, v/v) with 0.1% formic acid and 0.1 mM ammonium formate. The initial mobile phase was 30% solvent B at a flow rate of 300 μl/min. It was held for 2 min and then linearly increased to 100% solvent B for 23 min, followed by equilibration with 30% solvent B for 25 min. During the whole analysis, the samples were placed in an automatic sampler at 10°C. To avoid the influence caused by the fluctuation of the instrument detection signal, a random sequence was used for the continuous analysis of samples. Mass spectra were acquired using Q-Exactive Plus (Thermo Fisher Scientific, USA) in positive and negative modes. The following electrospray ionization parameters were optimized and preset for all measurements: 300°C, source temperature; 350°C, capillary temperature; 3,000 V, ion spray voltage; 50% S-Lens RF level; and m/z 200–1,800, instrument scan range.

LipidSearch software (Thermo Fisher Scientific) was used for peak recognition, peak extraction, and lipid identification (secondary identification) of lipid molecules and internal standard lipid molecules, which contain more than 30 lipid classes and more than 1,500,000 fragment ions in the database. Single-point internal standard calibrations were used to estimate the absolute concentrations of unique lipids identified by accurate MS, MS/MS spectral matching, and retention times (35). The main parameters were 5 ppm precursor tolerance, 5 ppm product tolerance, and 5% production threshold.

### Statistical Analyses

Differences in descriptive data were assessed using the chi-square test for categorical variables, and a non-parametric Mann–Whitney *U*-test or two-tailed Student's *t*-test was used for continuous variables. Lipid concentration is presented as mean ± standard deviation and was analyzed using two-tailed Student's *t*-test. Statistical significance was set at *P* < 0.05. Different lipid species were screened by fold change analysis, and the correlation between lipid concentrations and clinical parameters was analyzed by Spearman correlation. Potential biochemical markers were further evaluated using receiver operating characteristic (ROC) analysis, including transformation by a logistic regression model to the predicted probability scale. The area under the ROC curve (AUC) was used to assess the sensitivity and specificity of biomarkers.

## Results

### Clinical Characteristics of the Recruited Participants

A total of 24 women with BD and 30 female HCs were included in this study. No significant differences were found between the BD and HC groups in terms of age (*P* = 0.069), BMI (*P* = 0.984), and marital status (*P* = 0.321). The HAMD, HAMA, PANSS, and BRMS scale scores in the BD group were higher than those in the HC group (Table 1). Supplementary Table 1 shows the detailed clinical and demographic characteristics of the studied samples.

**Table 1:** Comparison of clinical characteristics data and symptom scale assessment between women with BD and HC.

**Parameter**	**HC (*n* = 30)**	**BD (*n* = 24)**	**t/Z/ χ^2^ value**	***P*** **-value**
**Sociodemographics**				
Age [years, M(*P*_25_, *P*_75_)]^b^	29.00 (25.00, 35.25)	32.50 (27.00, 41.50)	*Z* = −1.815	0.069
BMI (range)^a^	20.97 ± 2.88 (17.00–26.70)	20.94 ± 3.26 (14.68–27.99)	*t* = 0.038	0.970
Marital status (single/married/single after divorced) ^c^	12/17/1	6/15/3	*χ^2^* = 2.385	0.321
**Severity of depression symptoms and function assessment**				
HAMD [M(*P*_25_, *P*_75_)] ^b^	3.00 (2.00, 5.00)	18.00 (7.75, 26.75)	*Z* = −5.568	<0.001
HAMA [M (*P*_25_, *P*_75_)]^b^	4.50 (2.75, 6.25)	19.50 (7.25, 22.75)	*Z* = −4.859	<0.001
PANSS (range)^a^	35.63 ± 3.55 (30.00–42.00)	60.42 ± 18.66 (31.00–98.00)	*t* = −6.414	<0.001
BRMS [M (*P*_25_, *P*_75_)]^b^	2.00 (1.75, 3.00)	4.00 (3.00, 17.75)	*Z* = −3.906	<0.001

a *Student's t-test;*

b *Mann-Whitney U;*

c *Chi-square Tests; M, media; BMI, body mass index; Values are shown as mean ± standard deviation; HAMD, Hamilton Depression Scale; HAMA, Hamilton Anxiety Scale; PANSS, Positive and Negative Syndrome Scale; BRMS, Bech-Rafaelsen Mania Rating Scale*.

### Different Lipid Classes Between the BD and HC Groups and Their Correlation With Clinical Parameters

**Figure 1:**
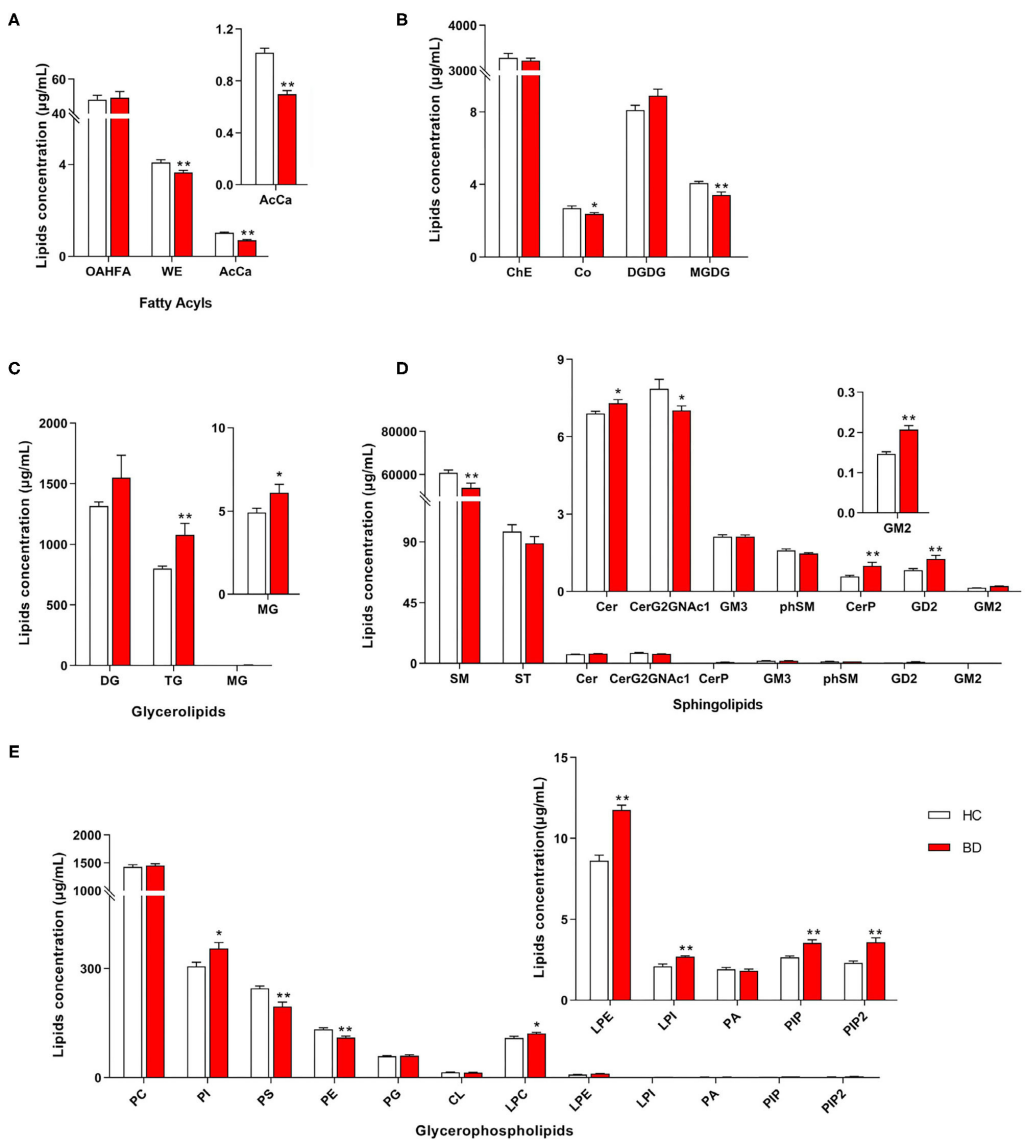
Different concentrations of lipid class between the BD and HC groups. **(A)** Fatty acyls, **(B)** ChE, Co, DGDG, and MGDG, **(C)** glycerolipids, **(D)** sphingolipids, and **(E)** glycerophospholipids. HC, healthy controls. Lipids showing low-level results have been enlarged in the insets. BD, bipolar disorder; OAHFA, (O-acyl)-1-hydroxy fatty acid; WE, wax esters; AcCa, acylcarnitine; DG, diglyceride; TG, triglyceride; MG, monoglyceride; SM, sphingomyelin; ST, sulfatide; Cer, ceramides; CerP, ceramides phosphate; phSM, phytosphingomyelin; ChE, cholesterol ester; Co, coenzyme; MGDG, monogalactosyldiacylglycerol; DGDG, digalactosyldiacylglycerol; PC, phosphatidylcholine; PI, phosphatidylinositol; PS, phosphatidylserine; PE, phosphatidylethanolamine; PG, phosphatidylglycerol; CL, cardiolipin; LPC, lysophosphatidylcholine; LPE, lysophosphatidylethanolamine; LPI, lysophosphatidylinositol; PA, phosphatidic acid; PIP, phosphatidylinositol phosphate; PIP2, phosphatidylinositol 4,5-bisphosphate. *P < 0.05 *vs*. HC; **P < 0.01 *vs*. HC.

A total of 31 lipid classes and 884 lipid species were identified in the samples of each group (Supplementary Table 2). Significant differences were observed in the concentrations of several classes of fatty acids (Figure 1A), saccharolipids, prenol lipids (Figure 1B), glycerolipids (Figure 1C), sphingolipids (Figure 1D), and glycerophospholipids (Figure 1E) between the HC and BD groups. The concentrations of wax esters (WEs) (*t* = 2.739, *P* = 0.008), acylcarnitine (AcCa) (*t* = 6.683, *P* < 0.001), sphingomyelin (SM) (*t* = 2.731, *P* = 0.009), CerG2GNAc1 (*t* = 2.062, *P* = 0.045), coenzyme (Co) (*t* = 2.183, *P* = 0.034), monogalactosyldiacylglycerol (MGDG) (*t* = 3.334, *P* = 0.002), phosphatidylserine (PS) (*t* = 3.595, *P* = 0.001), and phosphatidylethanolamine (PE) (*t* = 3.887, *P* < 0.001) were significantly lower in the BD group. By contrast, the concentrations of phosphatidylinositol (PI) (*t* = −2.618, *P* = 0.012), lysophosphatidylcholine (LPC) (*t* = −2.116, *P* = 0.040), lysophosphatidylethanolamine (LPE) (*t* = −6.675, *P* < 0.001), lysophosphatidylinositol (LPI) (*t* = −4.019, *P* < 0.001), PI phosphate (PIP) (*t* = −4.001, *P* < 0.001), PI 4,5-bisphosphate (PIP2) (*t* = −4.140, *P* < 0.001), ceramides (Cer) (*t* = −2.439, *P* = 0.019), Cer phosphate (CerP) (*t* = −2.837, *P* = 0.009), GD2 (*t* = −3.003, *P* = 0.005), GM2 (*t* = −5.060, *P* < 0.001), triglyceride (TG) (*t* = −2.891, *P* = 0.008), and monoglyceride (*t* = −2.082, *P* = 0.045) were significantly increased in the BD group.

**Figure 2:**
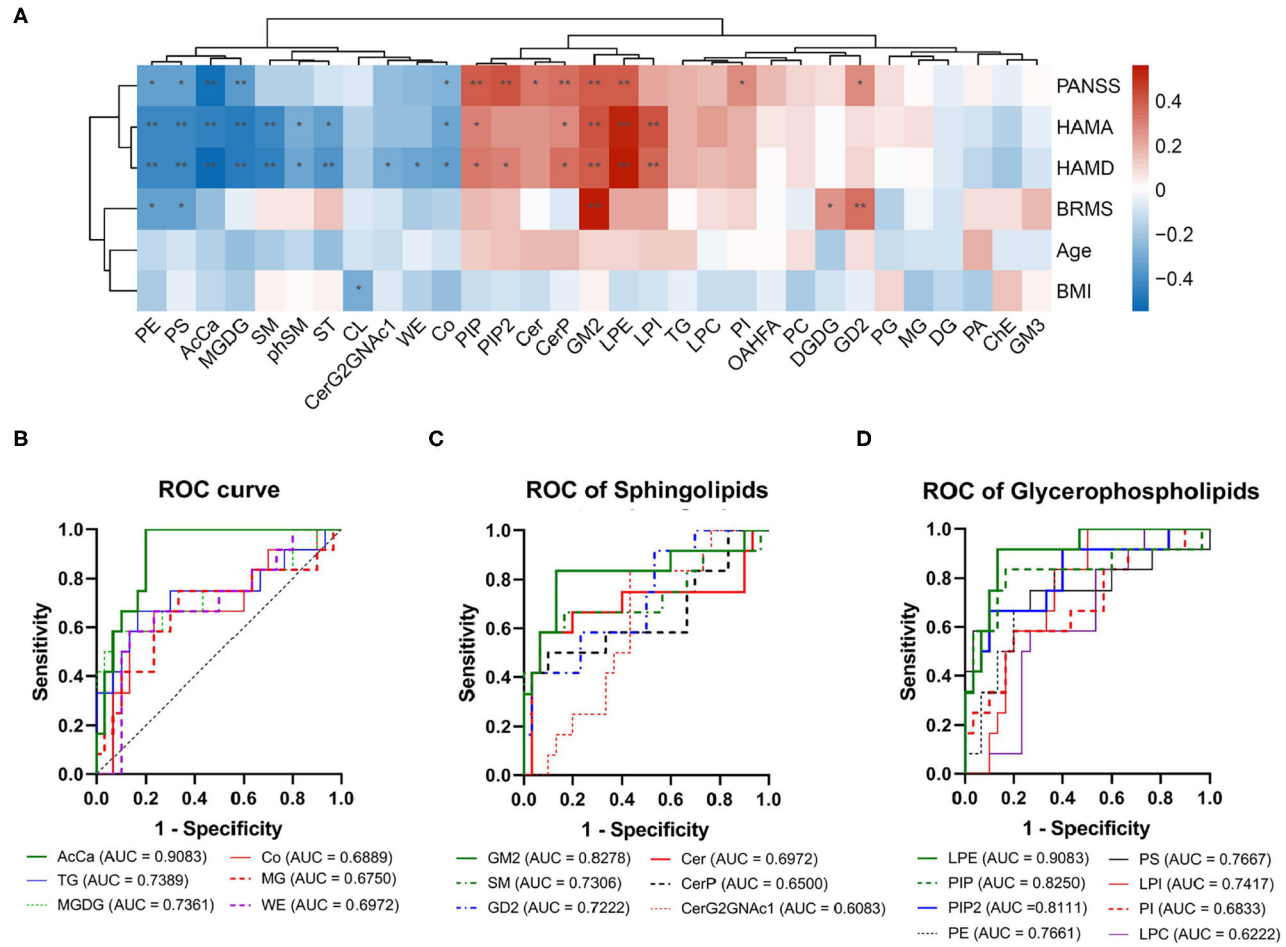
Correlation between clinical parameters and receiver operating characteristic (ROC) analysis for the different lipid classes. **(A)** Correlation analysis for levels of lipid classes and clinical parameters. The red squares show a positive correlation, and the blue squares show a negative correlation. *P < 0.05; **P < 0.01. **(B)** ROC of acylcarnitine, triglyceride, monogalactosyldiacylglycerol, coenzyme, monoglyceride, and wax esters. **(C)** ROC of sphingolipids. **(D)** ROC of glycerophospholipids.

Furthermore, the concentrations of PE, PS, AcCa, MGDG, and Co were negatively correlated with the total scores of PANSS, whereas the concentrations of PIP, PIP2, Cer, CerP, GM2, LPE, PI, and GD2 were positively correlated with the total scores of PANSS (Figure 2A). The concentrations of PE, PS, AcCa, MGDG, SM, phytosphingomyelin, and sulfatide were negatively correlated with the scores of both HAMA and HAMD, whereas the concentrations of PIP, CerP, GM2, LPE, and PI were positively correlated with the scores of both HAMA and HAMD. The concentrations of CerG2GNAc1, WE, and Co were negatively correlated with the scores of HAMD, whereas the concentrations of PIP2 were positively correlated with the scores of HAMD. The concentrations of Co were also negatively correlated with the scores of HAMA. Notably, the concentrations of PE and PS were negatively correlated with the scores of BRMS, whereas the concentrations of GM2, DGDG, and GD2 were positively correlated with the scores of BRMS (detailed data are displayed in Supplementary Table 3). Taken together, the concentrations of 8 and 12 lipids in the BD group, respectively, decreased and increased at the class level compared with those in the HC group. Moreover, changes in these lipid classes were related to the severity of psychotic, affective, and mania symptoms.

### ROC Analysis for Lipid Classes

ROC test analysis was performed to verify the specificity and predictive accuracy of the different lipid classes, which could be used to distinguish patients with BD from normal controls. As shown in Figure 2, the AUC of both AcCa and LPE was >0.9, thus indicating high reliability (Figures 2B,D). By contrast, GM2, SM, GD2, TG, MGDG, PIP, PIP2, PE, PS, and LPI showed moderate reliability (the AUC value was between 0.7 and 0.9; Figures 2B–D), thus suggesting that changes in AcCa and LPE might be potential markers for BD diagnosis at the class level.

### Different Carbon Chain Lengths and Degree of Saturation of Fatty Acid Between the BD and HC Groups

**Figure 3:**
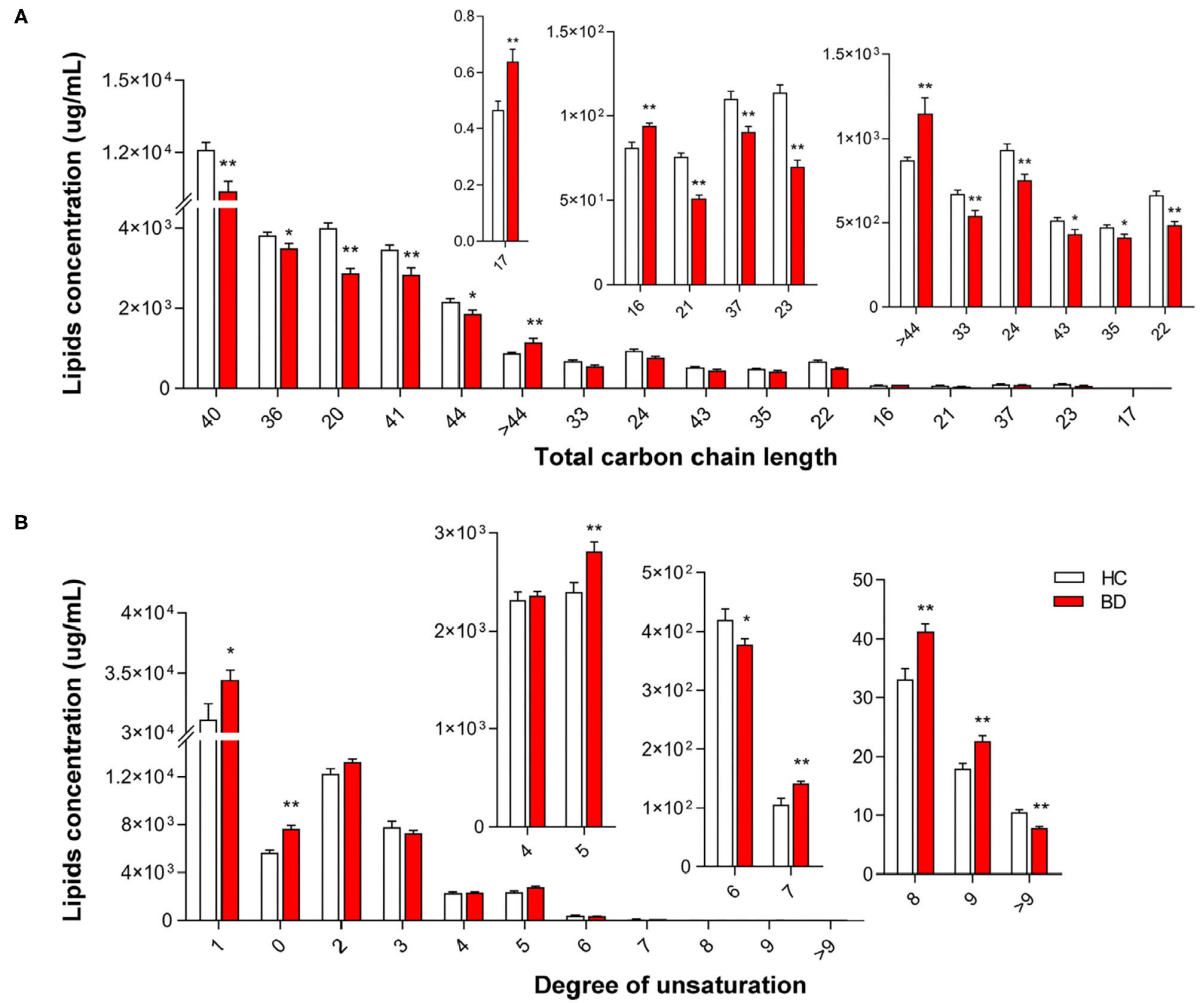
Groupwise alterations in the fatty acid composition of plasma lipids. Results of the analysis of fatty acyl composition by **(A)** chain length (number of carbons) and **(B)** degree of unsaturation. Lipids showing low-level results have been enlarged in the insets. *P < 0.05 *vs*. healthy controls (HC); **P < 0.01 *vs*. HC.

We observed significant alterations in the fatty acid chain profile of lipids between the HC and BD groups (Figure 3). The levels of long-chain fatty acids with 16 carbons, 17 carbons, and more than 44 carbons increased, whereas the levels of long-chain fatty acids with 20 to 44 carbons (20 carbons ≤ carbon length ≤ 44 carbons), except for 25–32 carbons, 34 carbons, 38 carbons, 39 carbons, and 42 carbons, decreased in the BD group (BD *vs*. HC, *P* < 0.05) (Figure 3A). Additionally, significant alterations in the degree of carbon chain saturation were observed between the HC and BD groups. In the BD group, the levels of saturated fatty acids, monounsaturated fatty acids, and polyunsaturated fatty acids with five double bonds, eight double bonds, and nine double bonds increased, whereas the levels of polyunsaturated fatty acids with six double bonds and more than nine double bonds decreased (BD *vs*. HC, *P* < 0.05) (Figure 3B). Furthermore, alterations in the concentrations of different lipid classes with different carbon chains and unsaturated fatty acids in the plasma of BD were also observed (Supplementary Figures 1, 2). Therefore, changes in plasma lipids in BD affected not only the lipid concentration but also the carbon chain length and degree of saturation of the fatty acid chain.

### Characteristic Lipid Species in the BD Group and Its Correlation With Clinical Parameters

**Figure 4:**
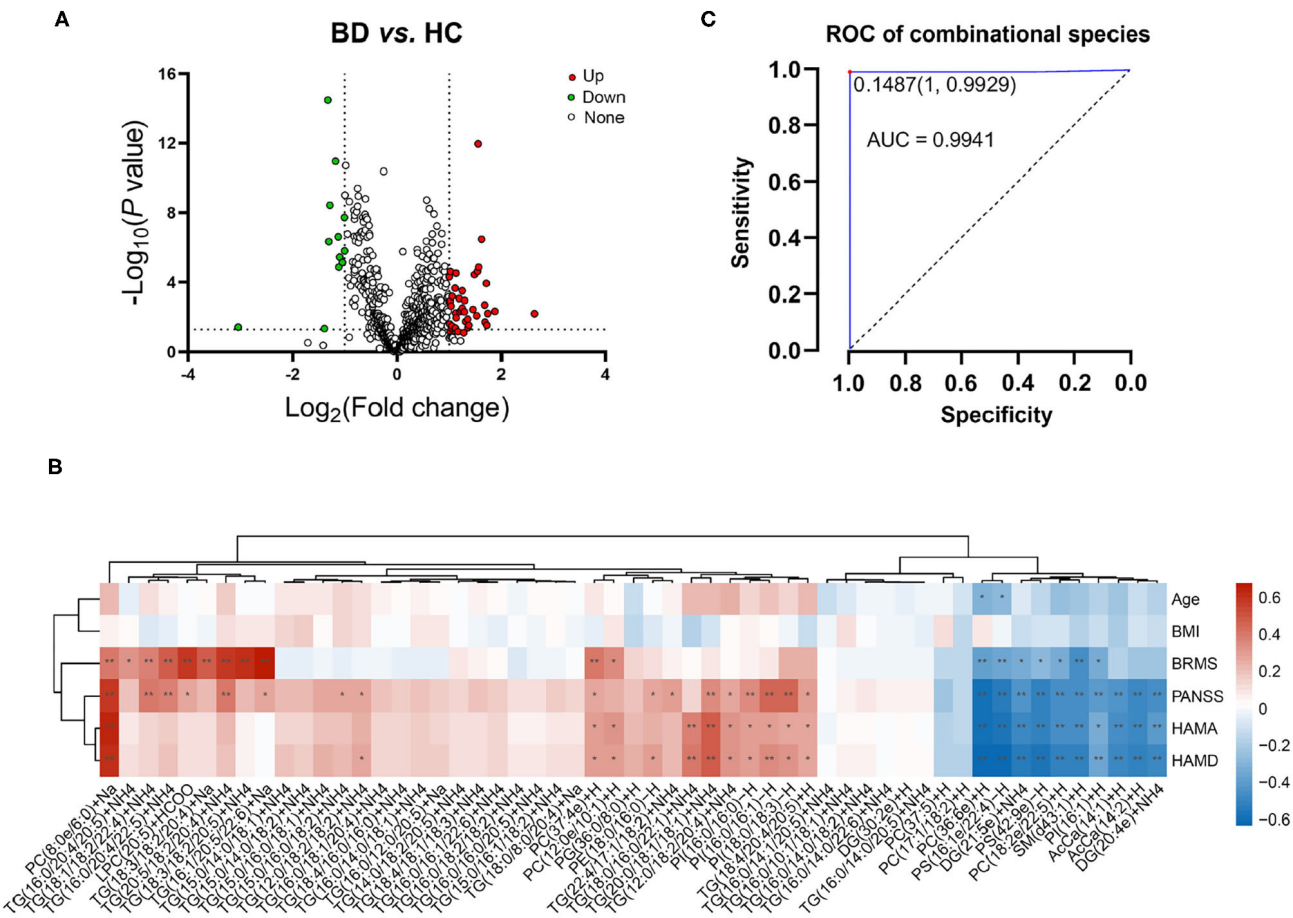
Characterization of lipid species in BD and their correlation with clinical parameters. **(A)** Volcano map revealing the decrease (green dots) and increase (red dots) in lipid species between patients with BD and the healthy controls. **(B)** Correlation between the clinical parameters and levels of lipid species in plasma. *P < 0.05; **P < 0.01. **(C)** Receiver operating characteristic of combinational molecules: PS(42:9e)-H, DG(21:5e)+NH_4_, PC(36:6e)+H, PC(8:0e/6:0)+Na, PS(16:1e/22:4)-H, TG(16:0/16:1/22:6)+NH_4_, TG(16:0/20:4/22:5)+NH_4_, and TG(22:4/17:1/18:2)+NH_4_, TG(18:0/8:0/20:4)+Na.

Lipidomic profiling further revealed changes in lipid concentrations between the BD and HC groups at the species level (Figure 4A). In general, in the BD group, the concentrations of 12 lipids, such as PC(37:5)+H, PS(16:1e/22:4)-H, and PS(42:9e)-H, decreased; by contrast, the levels of 43 lipids, such as TG(18:0/8:0/20:4)+Na, PI(16:0/16:1)-H, and PC(8:0e/6:0)+Na, increased (Table 2). A correlation analysis further showed that the concentrations of PC(17:1/18:2)+H, PC(36:6e)+H, PS(16:1e/22:4)-H, DG(21:5e)+NH_4_, PS(42:9e)-H, PC(18:2e/22:5)+H, SM(d43:1)+H, PI(16:1)+H, DG(20:4e)+NH_4_, AcCa(14:1)+H, and AcCa(14:2)+H were negatively correlated with the PANSS, HAMA, and HAMD scores (Figure 4B). By contrast, the concentrations of PC(8:0e/6:0)+Na, PC(37:4e)+H, TG(20:0/18:1/18:1)+NH_4_, TG(12:0/18:2/20:4)+NH_4_, PI(16:0/16:0)-H, PI(16:0/16:1)-H, PI(18:0/18:3)-H, and TG(18:4/20:4/20:5)+H were positively correlated with the PANSS, HAMA, and HAMD scores, thus suggesting that the changes in these lipid species were related to the severity of psychotic and affective symptoms. Notably, the concentrations of PC(17:1/18:2)+H, PC(36:6e)+H, PS(16:1e/22:4)-H, DG(21:5e)+NH_4_, PS(42:9e)-H, PC(18:2e/22:5)+H, SM(d43:1)+H, and PI(16:1)+H were negatively correlated with the BRMS scores, whereas the concentrations of PC(37:4e)+H, PC(12:0e/10:1)+H, PC(8:0e/6:0)+Na, TG(16:0/20:4/20:5)+NH_4_, TG(18:1/18:2/22:4)+NH_4_, TG(16:0/20:4/22:5)+NH_4_, LPC(20:5)+HCOO, TG(18:3/18:2/20:4)+Na, TG(20:5/18:2/20:4)+NH_4_, TG(18:3/18:2/20:5)+NH_4_, and TG(16:1/20:5/22:6)+Na were positively correlated with the BRMS scores (detailed data are displayed in Supplementary Table 4), thus indicating that the changes in these lipid molecules were related to mania symptoms.

**Table 2:** Altered concentrations of lipids species in the plasma between BD and HC group.

**LipidIon**	**LipidGroup**	**Class**	**Concentration (μg/mL)**		**Fold change**	***P*** **-value**
			**BD**	**HC**		
AcCa(14:2)+H	AcCa(14:2)+H	AcCa	0.029 ± 0.019	0.072 ± 0.032	0.404	<0.001
AcCa(14:1)+H	AcCa(14:1)+H	AcCa	0.038 ± 0.026	0.079 ± 0.032	0.485	<0.001
DG(20:4e)+NH4	DG(20:4e)+NH4	DG	0.427 ± 0.27	0.913 ± 0.39	0.468	<0.001
DG(21:5e)+NH4	DG(21:5e)+NH4	DG	3.142 ± 0.584	6.298 ± 2.267	0.499	<0.001
DG(30:2e)+H	DG(30:2e)+H	DG	12.385 ± 18.796	4.832 ± 4.948	2.563	0.039
LPC(20:5)+HCOO	LPC(20:5)+HCOO	LPC	0.417 ± 0.313	0.2 ± 0.086	2.081	0.001
PC(37:5)+H	PC(37:5)+H	PC	0.639 ± 0.259	5.257 ± 10.533	0.122	0.037
PC(17:1/18:2)+H	PC(35:3)+H	PC	0.106 ± 0.083	0.279 ± 0.403	0.381	0.044
PC(36:6e)+H	PC(36:6e)+H	PC	0.247 ± 0.044	0.617 ± 0.16	0.400	<0.001
PC(18:2e/22:5)+H	PC(40:7e)+H	PC	0.109 ± 0.065	0.238 ± 0.089	0.459	<0.001
PC(37:4e)+H	PC(37:4e)+H	PC	0.373 ± 0.383	0.159 ± 0.058	2.345	0.004
PC(12:0e/10:1)+H	PC(22:1e)+H	PC	0.019 ± 0.018	0.008 ± 0.003	2.458	0.001
PC(8:0e/6:0)+Na	PC(14:0e)+Na	PC	0.018 ± 0.006	0.006 ± 0.003	2.943	<0.001
PE(18:0/16:0)-H	PE(34:0)-H	PE	0.03 ± 0.018	0.014 ± 0.006	2.189	<0.001
PG(36:0/8:0)+H	PG(44:0)+H	PG	0.598 ± 0.65	0.244 ± 0.1	2.450	0.005
PI(16:1)+H	PI(16:1)+H	PI	0.131 ± 0.046	0.283 ± 0.15	0.462	<0.001
PI(16:0/16:0)-H	PI(32:0)-H	PI	1.116 ± 0.928	0.488 ± 0.26	2.285	0.001
PI(18:0/18:3)-H	PI(36:3)-H	PI	2.128 ± 1.583	0.718 ± 0.275	2.965	<0.001
PI(16:0/16:1)-H	PI(32:1)-H	PI	2.118 ± 1.912	0.645 ± 0.317	3.283	<0.001
PS(42:9e)-H	PS(42:9e)-H	PS	5.354 ± 1.28	13.043 ± 5.186	0.411	<0.001
PS(16:1e/22:4)-H	PS(38:5e)-H	PS	6.37 ± 2.367	14.416 ± 4.019	0.442	<0.001
SM(d43:1)+H	SM(d43:1)+H	SM	7.35 ± 3.898	14.73 ± 5.683	0.499	<0.001
TG(20:5/18:2/20:4)+NH4	TG(58:11)+NH4	TG	0.154 ± 0.093	0.077 ± 0.02	2.004	<0.001
TG(15:0/16:1/18:2)+NH4	TG(49:3)+NH4	TG	0.61 ± 0.698	0.303 ± 0.173	2.012	0.024
TG(18:0/16:0/22:1)+NH4	TG(56:1)+NH4	TG	0.624 ± 0.503	0.309 ± 0.105	2.017	0.002
TG(16:0/14:0/22:6)+NH4	TG(52:6)+NH4	TG	0.243 ± 0.345	0.12 ± 0.102	2.026	0.069
TG(16:0/20:4/20:5)+NH4	TG(56:9)+NH4	TG	0.318 ± 0.22	0.157 ± 0.12	2.030	0.001
TG(20:0/18:1/18:1)+NH4	TG(56:2)+NH4	TG	0.293 ± 0.165	0.144 ± 0.057	2.031	<0.001
TG(12:0/18:2/20:4)+NH4	TG(50:6)+NH4	TG	0.04 ± 0.031	0.02 ± 0.014	2.049	0.002
TG(16:0/18:2/18:2)+NH4	TG(52:4)+NH4	TG	6.011 ± 7.496	2.92 ± 2.711	2.058	0.041
TG(16:0/14:0/18:2)+NH4	TG(48:2)+NH4	TG	15.042 ± 18.52	7.25 ± 4.931	2.075	0.031
TG(16:0/10:1/18:1)+NH4	TG(44:2)+NH4	TG	0.087 ± 0.12	0.042 ± 0.102	2.077	0.142
TG(22:4/17:1/18:2)+NH4	TG(57:7)+NH4	TG	0.114 ± 0.083	0.053 ± 0.016	2.164	<0.001
TG(15:0/14:0/18:1)+NH4	TG(47:1)+NH4	TG	0.691 ± 0.885	0.318 ± 0.372	2.171	0.041
TG(18:4/18:1/18:3)+NH4	TG(54:8)+NH4	TG	0.672 ± 0.68	0.308 ± 0.125	2.179	0.006
TG(16:0/14:0/18:1)+NH4	TG(48:1)+NH4	TG	16.493 ± 16.966	7.515 ± 6.745	2.195	0.011
TG(16:0/14:1/20:5)+NH4	TG(50:6)+NH4	TG	0.069 ± 0.096	0.031 ± 0.051	2.247	0.067
TG(16:0/18:1/20:4)+NH4	TG(54:5)+NH4	TG	15.846 ± 16.598	6.881 ± 1.276	2.303	0.005
TG(14:0/18:2/20:5)+NH4	TG(52:7)+NH4	TG	0.502 ± 0.503	0.211 ± 0.087	2.377	0.003
TG(18:3/18:2/20:4)+Na	TG(56:9)+Na	TG	0.029 ± 0.023	0.012 ± 0.004	2.379	<0.001
TG(15:0/14:0/18:2)+NH4	TG(47:2)+NH4	TG	0.174 ± 0.275	0.072 ± 0.13	2.426	0.078
TG(16:0/12:0/20:5)+Na	TG(48:5)+Na	TG	0.07 ± 0.084	0.028 ± 0.039	2.494	0.018
TG(18:3/18:2/20:5)+NH4	TG(56:10)+NH4	TG	0.096 ± 0.122	0.037 ± 0.02	2.561	0.013
TG(15:0/16:0/18:2)+NH4	TG(49:2)+NH4	TG	1.935 ± 2.866	0.745 ± 0.402	2.599	0.029
TG(15:0/16:0/18:1)+NH4	TG(49:1)+NH4	TG	3.358 ± 3.735	1.222 ± 0.782	2.748	0.004
TG(18:4/20:4/20:5)+H	TG(58:13)+H	TG	0.053 ± 0.04	0.019 ± 0.009	2.805	<0.001
TG(16:1/20:5/22:6)+Na	TG(58:12)+Na	TG	0.053 ± 0.068	0.018 ± 0.007	2.887	0.008
TG(18:1/18:2/22:4)+NH4	TG(58:7)+NH4	TG	1.383 ± 1.042	0.474 ± 0.236	2.918	<0.001
TG(16:0/20:4/22:5)+NH4	TG(58:9)+NH4	TG	0.165 ± 0.099	0.054 ± 0.03	3.079	<0.001
TG(16:0/16:1/22:6)+NH4	TG(54:7)+NH4	TG	3.055 ± 3.528	0.951 ± 0.279	3.213	0.002
TG(16:0/16:0/20:5)+NH4	TG(52:5)+NH4	TG	1.288 ± 1.901	0.4 ± 0.587	3.223	0.019
TG(16:0/14:0/20:5)+NH4	TG(50:5)+NH4	TG	0.386 ± 0.653	0.117 ± 0.078	3.304	0.029
TG(18:4/16:0/16:0)+NH4	TG(50:4)+NH4	TG	2.835 ± 3.723	0.846 ± 0.739	3.349	0.006
TG(12:0/18:2/18:2)+NH4	TG(48:4)+NH4	TG	0.248 ± 0.33	0.067 ± 0.046	3.683	0.005
TG(18:0/8:0/20:4)+Na	TG(46:4)+Na	TG	0.08 ± 0.127	0.013 ± 0.023	6.178	0.006

### Potential Lipid Biomarkers of BD

The ROC analysis revealed that 38 lipid species, such as TG(18:1/18:2/22:4)+NH_4_, AcCa(14:2)+H, and PC(18:2e/22:5)+H, showed moderate or high reliability (AUC >0.7; Supplementary Table 5). Notably, the AUC of PS(42:9e)-H, DG(21:5e)+NH_4_, PC(36:6e)+H, PC(8:0e/6:0)+Na, PS(16:1e/22:4)-H, TG(16:0/16:1/22:6)+NH_4_, TG(16:0/20:4/22:5)+NH_4_, TG(22:4/17:1/18:2)+NH_4_, and TG(18:0/8:0/20:4)+Na, respectively, which covered different lipid species and demonstrated different change tendencies in BD subjects, was higher than 0.9 and was defined as a combinational biomarker. We then transformed the concentrations of these nine lipids to the predicted probability scale by using a logistic regression model, and the results showed high sensitivity and specificity (AUC = 0.994, 95% CI: 0.9826–1, *P* < 0.001) (Figure 4C). Therefore, the characteristic changes in these species may be a peripheral diagnostic biomarker of BD.

## Discussion

We investigated the characteristics of peripheral lipid composition and potential lipid biomarkers for female BD patients by comprehensive lipid profiling and quantitative validation based on LC–MS/MS. The lipidomic analysis showed that alterations in several lipid classes, such as AcCa and LPE, were remarkably changed in patients with BD, which were positively or negatively correlated with the severity of psychotic, affective, or mania symptoms. We also found that the composition of different carbon chain lengths and degrees of saturation of fatty acids in BD also differed from those in HCs. Moreover, we screened 55 lipid molecules with significant differences and investigated their correlations with clinical parameters. Finally, we identified a plasma biomarker set that comprised several lipids, including PS(42:9e)-H, DG(21:5e)+NH_4_, PC(36:6e)+H, PC(8:0e/6:0)+Na, PS(16:1e/22:4)-H, TG(16:0/16:1/22:6)+NH_4_, TG(16:0/20:4/22:5)+NH_4_, TG(22:4/17:1/18:2)+NH_4_, and TG(18:0/8:0/20:4)+Na, which can be used to differentiate female patients with BD from HCs.

The biological functions of lipids are associated with multiple processes, including inflammation, apoptosis, proliferation, and differentiation (36, 37), and disturbances in lipid metabolism have been demonstrated in various neuropsychiatric diseases (38, 39). Although changes in plasma lipids have also been observed in mental disorders, including schizophrenia, depression, and BD (40, 41), we believe that the current study is the first study to quantitatively compare plasma lipid composition in adult women with BD vs. HCs. Herein we found that the levels of SM, AcCa, PE, PS, and Co decreased in BD and were negatively correlated with the severity of psychotic, affective, or mania symptoms. Previous studies also found that plasma SM was negatively correlated with depressive symptoms (42), and lower concentrations of AcCa and PE were also observed in the plasma of BD patients than that of HCs (43–45). Considering that PS biosynthesis *via* the serine base exchange reaction using PE and PS can be converted to PE by PS decarboxylase in the mitochondria (46), the decrease in PS levels may be related to the reduction of PE. Similarly, although a decrease in plasma CoQ10 has not been reported in BD, adjuvant CoQ10 might be considered a safe and effective strategy for the treatment of patients with BD during their depressive phase (47). On the other hand, we also found that the levels of Cer, CerP, PI, LPE, LPI, PIP, and PIP2 increased in BD and were positively correlated with the severity of psychotic, affective, or mania symptoms. Consistent with this finding, previous studies have found that plasma Cer was substantially increased in patients with major depression and BD irrespective of the severity of symptoms in the current episode (48). PIP2 was increased in the platelet membrane of drug-free depressed bipolar patients, whereas therapeutic doses of lithium significantly decreased the platelet membrane PIP2 levels *in vivo* in BD subjects (49, 50). Moreover, although changes in plasma LPE and PI have not been reported in BD, a previous work reported that LPE and PI were remarkably increased in MDD and showed pronounced positive relationships with depression severity (51).

Notably, the ROC analysis further found that AcCa and LPE showed high reliability (AUC > 0.9) in distinguishing BD from HCs. AcCa is a class of metabolites that are formed from the transfer of the acyl group of fatty acyl-CoA to carnitine (52) and is involved in mitochondrial function and energy metabolism, antioxidative functions, and neuroprotection (53, 54). LPE is a lyso-type metabolite of PE produced *via* a phospholipase A-type reaction, and only a limited number of studies have reported that serum LPE was significantly decreased in patients with migraine and non-alcoholic fatty liver disease (55, 56). Furthermore, a decrease in MGDG and an increase in GD2 and GM2 were observed in the plasma of BD patients. Disrupted GM2/GD2 synthase exhibited neurodegeneration in the nervous system (57), and GM2 elevation was associated with glial activation in the injured developing brain (58). MGDGs show strong anti-inflammatory properties (59, 60). Taken together, the composition of plasma lipids was altered in women with BD. Consistent with the pathophysiology of BD, most of these lipid classes are related to oxidative stress, inflammatory responses, neuroprotection, and mitochondrial dysfunction (61–63).

In addition to observing changes in lipid class levels, we also compared the carbon chain lengths and unsaturated bonds of fatty acids in different classes between the two groups. The carbon chain length and degree of saturation of fatty acids influence the biophysical properties of lipids; longer and more fully saturated chains are correlated with more extensive stabilizing lipid–protein and lipid–lipid interactions and a higher free energy barrier for protein dissociation and lipoprotein fusion (64)—for instance, the modulation of the composition of fatty acyl chains and degree of saturation of membrane lipids can potentially affect neuronal functioning, at least in part, *via* the altered function of membrane-bound proteins (65, 66). The present study found that long-chain and polyunsaturated fatty acids were mainly decreased in BD. Moreover, we compared the concentrations of different lipid classes with different carbon chains and unsaturated fatty acids in the plasma of BD vs. HC. Although the potential mechanism for these differences is not clear, this result sheds a new light on existing data on the differences in lipid class structure and function in women with BD.

In addition to analyzing the composition of lipid classes that are characterized in BD, we further investigated the changes in lipids at the species level and identified which lipid biomarkers could discriminate BD for further development as a potential diagnostic tool. We observed 55 lipid species with significant differences and identified a signature of 9 lipids that could distinguish patients with BD from HCs (AUC = 0.994). We believe that this method has the potential to be used as a different diagnostic tool for distinguishing BD from HC on the basis of the subjects' plasma lipid composition and could fill the clinical need for better quantitative diagnostic tools to optimize the initial treatment approach. However, previous studies on BD and lipids have identified several lipids as potential BD biomarkers—for example, a preliminary study (15 BD patients and 21 healthy subjects) on BD and serum lipids found that PI(40:3), PG(32:4)-OH, and TG(42:3) showed high reliability (AUC > 0.9) in distinguishing BD type I from HCs, whereas PE(42:5), PA(48:8)-OH, and PA(44:4) showed moderate reliability (AUC > 0.8) in distinguishing BD type I from HCs (32). Another large-sample study (67 patients with unipolar disorder or BDs and 405 healthy subjects) used targeted lipidomics, such as C24:1Cer, C24:1GluCer, and C24LacCer, and found that the differences between MDD and BD patients *versus* controls mainly originated from Cer and their hexosyl metabolites (48). However, we did not measure either of these lipids in our untargeted lipidomics analysis. This discrepancy might be partly due to the different methods for evaluating lipidomics (*i.e*., different types of lipids were quantified) and the different age/sex distributions. However, the results of lipid composition at the class and species levels were not completely consistent—for example, the TG levels increased in BD with no significant correlation with clinical symptoms, whereas the levels of TG(16:0/16:1/22:6)+NH_4_, TG(16:0/20:4/22:5)+NH_4_, TG(22:4/17:1/18:2)+NH_4_, and TG(18:0/8:0/20:4)+Na were changed in BD, had a significant correlation with the severity of clinical symptoms, and exhibited high reliability. Other species including TG(18:1/18:2/22:4)+NH_4_, TG(18:4/20:4/20:5)+H, TG(12:0/18:2/18:2)+NH_4_, TG(16:0/14:1/20:5)+NH_4_, TG(16:0/20:4/20:5)+NH_4_, TG(20:0/18:1/18:1)+NH_4_, and TG(15:0/16:0/18:1)+NH_4_ also showed moderate reliability for BD diagnosis. Therefore, future studies using consistent methods for a more comprehensive coverage of lipid metabolites with larger sample sizes would be instrumental for clarifying and confirming specific lipid species that may be altered in BD.

Furthermore, although the dysfunction of lipid metabolism in peripheral blood may contribute to the etiopathology of BD (67) and given that the use of nutritional and other treatments aimed at modulating the function and regulation of lipids, such as phospholipids and sphingolipids, represents a means to improve or prevent BD in individuals suffering from mood disorders (68–70), the lipid changes in the brain tissue and plasma of patients with BD are not entirely consistent, and the crosstalk between peripheral and brain lipid homeostasis is still unclear—for example, we found that PE and PS decreased in the plasma of BD patients; however, a previous study showed that there was no significant difference in total PS, PE, and PC in the postmortem hippocampus of patients with BD and the HCs (71). An analysis of brain lipids and associated enzymes in the cerebrospinal fluid might be used to investigate the central characteristics of lipid status and provide further insight into the relationship between lipid homeostasis and the clinical symptoms of BD (37). Moreover, female patients with unipolar depression were excluded from the current study. Whether the potential lipids could distinguish bipolar and unipolar depression remains to be further investigated, and the potential molecular mechanism involved in the pathophysiology of female BD also remains to be determined. Although lipid levels were quantitatively analyzed in this study, the internal mix standards only contained 14 lipids. Considering that the content of the substance was calculated using the response abundance ratio (peak area ratio) between the substance and the internal standard, it does not really reflect the absolute content of all substances and cannot be compared with the clinical reference range of the lipid content of blood samples.

In summary, our study revealed distinct changes in the lipid composition of female BD patients, identified 55 lipids with significant differences, investigated their correlation with clinical parameters, and screened 9 species as complementary biomarkers for the diagnosis of BD. These data provide a further understanding of abnormal lipid metabolism and suggest that specific lipid species can be used to distinguish female patients with BD from normal controls. However, the number of recruited participants in this study was relatively small, and the correlation coefficient between the clinical scale score and the lipid content was low. Although it was consistent with the results of previous studies (42, 51), it suggested that the strength of the relationship was insufficient and that there might be a shortage of lipid markers for the prediction of symptom severity. Furthermore, this study was a cross-sectional study, and the plasma samples were collected only at baseline, thus making it difficult to establish a causal association between changes in lipid composition and disease remission. A discovery and validation cohort study with a larger sample size should be conducted in the future to verify the potential use of these lipid markers.

## Data Availability Statement

The original contributions presented in the study are included in the article/Supplementary Materials, further inquiries can be directed to the corresponding authors.

## Ethics Statement

The study protocol was approved by Chinese Registered Clinical Trial Ethics Committee (Approval Number: ChiECRCT20200090) and was registered with the Chinese Clinical Trial Registry (Registration number ChiCTR2000032118). The patients/participants provided their written informed consent to participate in this study.

## Author Contributions

LG, TZ, RL, JD, and J-bY designed the data collection tool and were responsible for data collection and clinical evaluation. Z-qC was responsible for data management. Z-wP and Q-rT financed and designed the study and supervised the data collection and analysis. Y-HC, FX, and RL analyzed the data. LG and TZ wrote the first draft with Z-wP and Q-rT. All other authors provided the data, reviewed the results, and contributed to the final draft of the report.

## Funding

This study was funded by the National Natural Science Foundation of China (Grant Nos. 81630032, 82171512, and 82101594).

## Conflict of Interest

The authors declare that the research was conducted in the absence of any commercial or financial relationships that could be construed as a potential conflict of interest.

## Publisher's Note

All claims expressed in this article are solely those of the authors and do not necessarily represent those of their affiliated organizations, or those of the publisher, the editors and the reviewers. Any product that may be evaluated in this article, or claim that may be made by its manufacturer, is not guaranteed or endorsed by the publisher.

## Supplementary Material

Supplementary Figure 1 Comparison of the concentrations of lipids with different carbon chain lengths from the fatty acyls of the BD and HC groups. Lipids showing low-level results have been enlarged in the insets. **(A)** AcCa; **(B)** WE; **(C)** MGDG; **(D)** TG; **(E)** CerP; **(F)** SM; **(G)** Cer; **(H)** CerG2GNAc1; **(I)** PI, PIP and PIP2; **(J)** PS; **(K)** PE; **(L)** LPE and LPI; and **(M)** MG. ^*^*P* < 0.05; ^**^*P* < 0.01.

Supplementary Figure 2 Comparison of the concentrations of lipids with different degrees of saturation from the fatty acyls of the BD and HC groups. Lipids showing low-level results have been enlarged in the insets. **(A)** AcCa and WE; **(B)** SM, CerG2GNAc1, Cer and CerP; **(C)** PI, PS and PE; **(D)** MGDG; **(E)** LPC, LPE, LPI, PIP and PIP2; and **(F)** TG and MG. ^*^*P* < 0.05; ^**^*P* < 0.01.

Supplementary Table 1 Clinical information of enrolled individuals.

Supplementary Table 2 Original data of lipidomics.

Supplementary Table 3 Correlation of clinical parameters and the concentration of lipid class (spearman correlation).

Supplementary Table 4 Correlation of clinical parameters and the concentration of altered lipids at species level (spearman correlation).

Supplementary Table 5 ROC analysis for altered lipids at species level.

## Abbreviations

BD: bipolar disorder
LC: liquid chromatography
MS: Mass Spectrometry
DSM-5: fifth edition of the *Diagnostic and Statistical Manual of Mental Disorders*
HAMD: Hamilton Depression Rating Scale
HAMA: Hamilton Anxiety Scale
BMI: Body Mass Index
PANSS: Positive and Negative Syndrome Scale
BRMS: Bech–Rafaelsen Mania Rating Scale
ROC: Receiver Operating Characteristic
AUC: area under the ROC curve
HC: Healthy Controls
WE: Wax Ester
AcCa: acylcarnitine
DG: diglyceride
TG: triglyceride
MG: monoglyceride
SM: sphingomyelin
Cer: ceramides
CerP: ceramide phosphate
ChE: cholesterol ester
Co: coenzyme
MGDG: monogalactosyldiacylglycerol
DGDG: digalactosyldiacylglycerol
PC: phosphatidylcholine
PI: phosphatidylinositol
PS: phosphatidylserine
PE: phosphatidylethanolamine
PG: phosphatidylglycerol
CL: cardiolipin
LPC: lysophosphatidylcholine
LPE: lysophosphatidylethanolamine
LPI: lysophosphatidylinositol
PA: phosphatidic acid
PIP: phosphatidylinositol phosphate
PIP2, phosphatidylinositol 4: 5-bisphosphate.
