# Supplementary figures and images for: Alterations in the Plasma Lipidome of Adult Women With Bipolar Disorder: A Mass Spectrometry-Based Lipidomics Research
Source: Front Psychiatry. 2022 Mar 21;13:802710. doi: 10.3389/fpsyt.2022.802710 (PMC8978803; doi:10.3389/fpsyt.2022.802710)

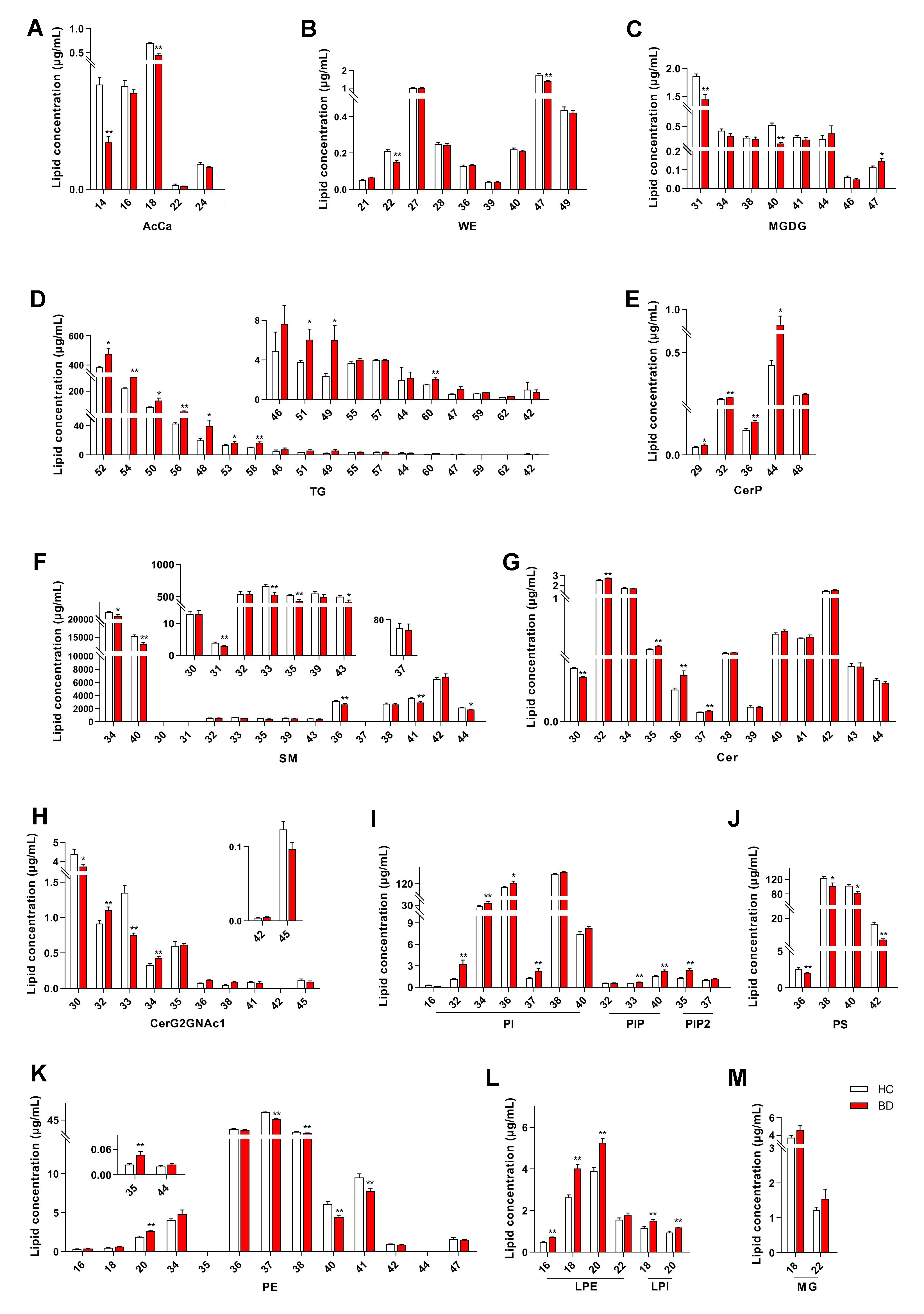

Supplement: Supplementary Figure 1 — Comparison of the concentrations of lipids with different carbon chain lengths from the fatty acyls of the BD and HC groups. Lipids showing low-level results have been enlarged in the insets. (A) AcCa; (B) WE; (C) MGDG; (D) TG; (E) CerP; (F) SM; (G) Cer; (H) CerG2GNAc1; (I) PI, PIP and PIP2; (J) PS; (K) PE; (L) LPE and LPI; and (M) MG. *P < 0.05; **P < 0.01. [file Image_1.jpeg]

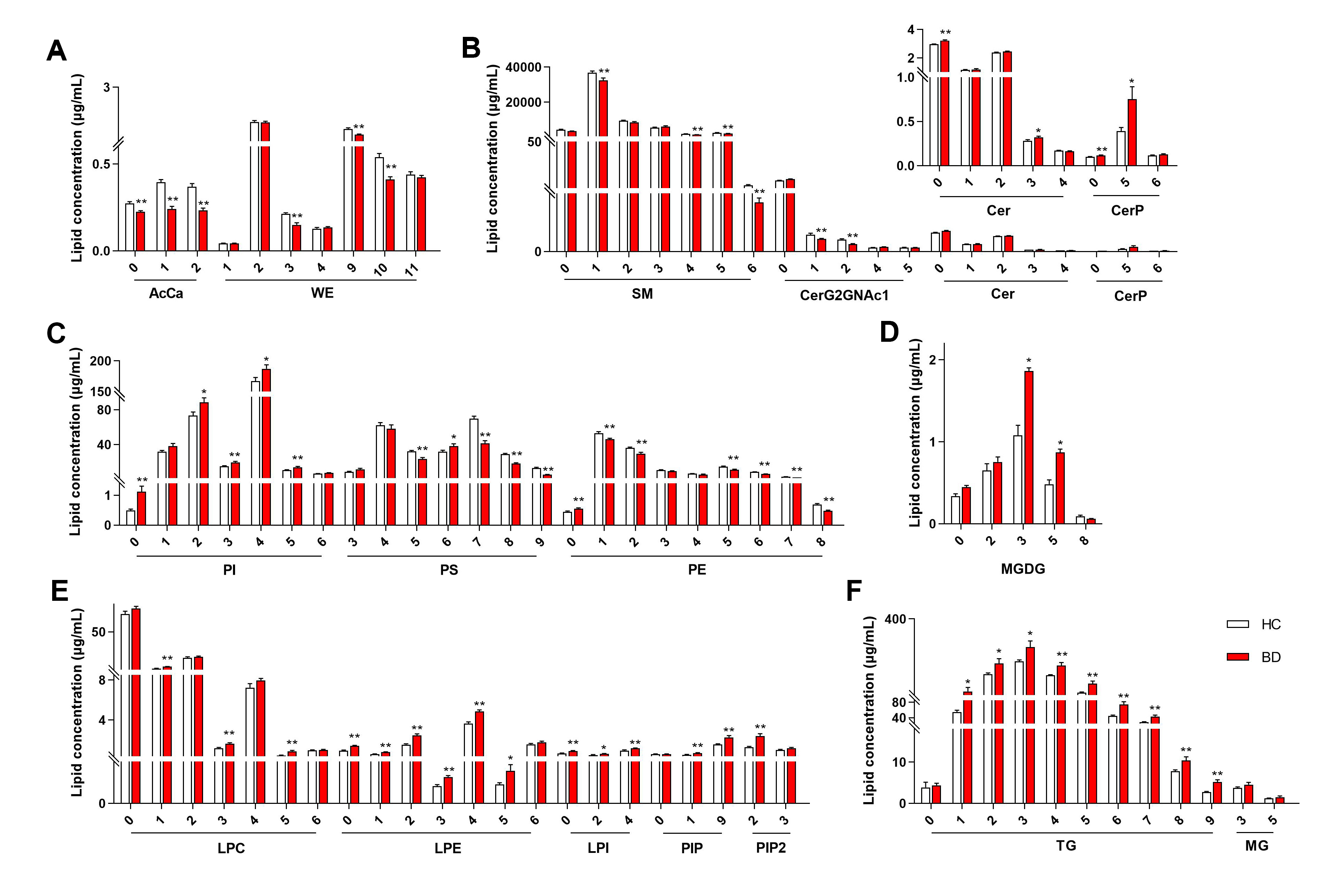

Supplement: Supplementary Figure 2 — Comparison of the concentrations of lipids with different degrees of saturation from the fatty acyls of the BD and HC groups. Lipids showing low-level results have been enlarged in the insets. (A) AcCa and WE; (B) SM, CerG2GNAc1, Cer and CerP; (C) PI, PS and PE; (D) MGDG; (E) LPC, LPE, LPI, PIP and PIP2; and (F) TG and MG. *P < 0.05; **P < 0.01. [file Image_2.jpeg]
