# Supplementary material for: Alterations in the Plasma Lipidome of Adult Women With Bipolar Disorder: A Mass Spectrometry-Based Lipidomics Research
Source: Front Psychiatry. 2022 Mar 21;13:802710. doi: 10.3389/fpsyt.2022.802710 (PMC8978803; doi:10.3389/fpsyt.2022.802710)
Supplement: Supplementary Table 3 — Correlation of clinical parameters and the concentration of lipid class (spearman correlation). [file Table_3.doc]

Supplemental table 3. Correlation of clinical parameters and the concentration of lipid class (spearman correlation).

| Class | Age | | BMI | | HAMA | | HAMD | | PANSS | | BRMS | |
| --- | --- | --- | --- | --- | --- | --- | --- | --- | --- | --- | --- | --- |
|  | *r* value | *P* value | *r* value | *P* value | *r* value | *P* value | *r* value | *P* value | *r* value | *P* value | *r* value | *P* value |
| AcCa | -0.170 | 0.218 | -0.145 | 0.295 | -0.454 | 0.001 | -0.548 | 0.000 | -0.521 | 0.000 | -0.237 | 0.085 |
| OAHFA | 0.023 | 0.871 | -0.043 | 0.759 | 0.060 | 0.664 | 0.017 | 0.903 | 0.162 | 0.240 | 0.005 | 0.970 |
| WE | -0.059 | 0.672 | -0.154 | 0.265 | -0.235 | 0.088 | -0.305 | 0.025 | -0.255 | 0.063 | -0.172 | 0.213 |
| MG | -0.098 | 0.482 | -0.209 | 0.130 | 0.086 | 0.538 | 0.025 | 0.859 | 0.024 | 0.864 | -0.027 | 0.845 |
| TG | 0.119 | 0.392 | -0.040 | 0.773 | 0.184 | 0.183 | 0.186 | 0.178 | 0.186 | 0.178 | 0.018 | 0.897 |
| DG | -0.090 | 0.515 | -0.146 | 0.293 | -0.062 | 0.657 | -0.062 | 0.655 | -0.049 | 0.727 | -0.095 | 0.492 |
| CL | -0.084 | 0.544 | -0.296 | 0.030 | -0.168 | 0.226 | -0.171 | 0.216 | -0.076 | 0.585 | -0.074 | 0.596 |
| LPC | -0.016 | 0.908 | -0.126 | 0.363 | 0.244 | 0.076 | 0.184 | 0.184 | 0.182 | 0.189 | 0.087 | 0.533 |
| LPE | 0.102 | 0.464 | -0.125 | 0.368 | 0.517 | 0.000 | 0.546 | 0.000 | 0.392 | 0.003 | 0.227 | 0.100 |
| LPI | 0.127 | 0.359 | -0.088 | 0.529 | 0.412 | 0.002 | 0.380 | 0.005 | 0.216 | 0.117 | 0.213 | 0.122 |
| PA | 0.188 | 0.174 | -0.179 | 0.194 | -0.087 | 0.533 | -0.141 | 0.308 | 0.048 | 0.732 | 0.089 | 0.522 |
| PC | 0.092 | 0.508 | -0.192 | 0.165 | 0.091 | 0.514 | 0.089 | 0.522 | 0.109 | 0.434 | -0.042 | 0.762 |
| PE | -0.132 | 0.342 | -0.193 | 0.162 | -0.421 | 0.002 | -0.423 | 0.001 | -0.335 | 0.013 | -0.335 | 0.013 |
| PG | -0.111 | 0.425 | 0.108 | 0.439 | 0.059 | 0.671 | -0.006 | 0.965 | 0.087 | 0.532 | -0.157 | 0.257 |
| PI | 0.012 | 0.929 | -0.114 | 0.412 | 0.201 | 0.146 | 0.198 | 0.151 | 0.276 | 0.043 | 0.104 | 0.454 |
| PIP | 0.134 | 0.333 | -0.112 | 0.422 | 0.310 | 0.022 | 0.334 | 0.014 | 0.390 | 0.004 | 0.171 | 0.216 |
| PIP2 | 0.150 | 0.279 | -0.085 | 0.539 | 0.265 | 0.053 | 0.299 | 0.028 | 0.411 | 0.002 | 0.144 | 0.300 |
| PS | -0.090 | 0.517 | -0.054 | 0.701 | -0.416 | 0.002 | -0.420 | 0.002 | -0.331 | 0.015 | -0.335 | 0.013 |
| Co | -0.104 | 0.452 | -0.230 | 0.094 | -0.311 | 0.022 | -0.330 | 0.015 | -0.276 | 0.043 | -0.153 | 0.270 |
| DGDG | -0.184 | 0.182 | -0.092 | 0.510 | -0.012 | 0.934 | -0.008 | 0.951 | 0.088 | 0.528 | 0.270 | 0.049 |
| MGDG | -0.226 | 0.100 | -0.177 | 0.200 | -0.481 | 0.000 | -0.474 | 0.000 | -0.351 | 0.009 | -0.039 | 0.779 |
| ChE | -0.073 | 0.601 | 0.142 | 0.306 | -0.152 | 0.273 | -0.212 | 0.124 | -0.048 | 0.733 | 0.077 | 0.580 |
| Cer | 0.178 | 0.198 | -0.114 | 0.412 | 0.267 | 0.051 | 0.260 | 0.058 | 0.338 | 0.012 | -0.005 | 0.973 |
| CerG2GNAc1 | -0.107 | 0.442 | -0.082 | 0.557 | -0.217 | 0.116 | -0.297 | 0.029 | -0.216 | 0.117 | -0.091 | 0.511 |
| CerP | 0.172 | 0.213 | -0.044 | 0.750 | 0.282 | 0.039 | 0.344 | 0.011 | 0.349 | 0.010 | -0.068 | 0.625 |
| GD2 | 0.082 | 0.556 | -0.055 | 0.692 | 0.084 | 0.547 | 0.099 | 0.478 | 0.279 | 0.041 | 0.357 | 0.008 |
| GM2 | 0.097 | 0.485 | 0.037 | 0.788 | 0.413 | 0.002 | 0.397 | 0.003 | 0.389 | 0.004 | 0.561 | 0.000 |
| GM3 | -0.067 | 0.628 | 0.048 | 0.732 | -0.050 | 0.720 | -0.088 | 0.529 | 0.011 | 0.935 | 0.176 | 0.203 |
| phSM | -0.139 | 0.315 | 0.019 | 0.891 | -0.300 | 0.027 | -0.305 | 0.025 | -0.154 | 0.266 | 0.071 | 0.612 |
| SM | -0.187 | 0.176 | 0.031 | 0.822 | -0.429 | 0.001 | -0.452 | 0.001 | -0.157 | 0.256 | 0.066 | 0.638 |
| ST | -0.238 | 0.083 | 0.043 | 0.755 | -0.339 | 0.012 | -0.375 | 0.005 | -0.094 | 0.501 | 0.150 | 0.278 |
