# Supplementary material for: Alterations in the Plasma Lipidome of Adult Women With Bipolar Disorder: A Mass Spectrometry-Based Lipidomics Research
Source: Front Psychiatry. 2022 Mar 21;13:802710. doi: 10.3389/fpsyt.2022.802710 (PMC8978803; doi:10.3389/fpsyt.2022.802710)
Supplement: Supplementary Table 4 — Correlation of clinical parameters and the concentration of altered lipids at species level (spearman correlation). [file Table_4.doc]

Supplemental table 4. Correlation of clinical parameters and the concentration of altered lipids at species level (spearman correlation).

| Species | Age | | BMI | | HAMA | | HAMD | | PANSS | | BRMS | |
| --- | --- | --- | --- | --- | --- | --- | --- | --- | --- | --- | --- | --- |
|  | *r* value | *P* value | *r* value | *P* value | *r* value | *P* value | *r* value | *P* value | *r* value | *P* value | *r* value | *P* value |
| AcCa(14:2)+H | -0.132 | 0.341 | -0.089 | 0.523 | -0.478 | 0.000 | -0.514 | 0.000 | -0.491 | 0.000 | -0.218 | 0.113 |
| AcCa(14:1)+H | -0.215 | 0.118 | -0.197 | 0.153 | -0.456 | 0.001 | -0.494 | 0.000 | -0.425 | 0.001 | -0.169 | 0.223 |
| DG(20:4e)+NH4 | -0.183 | 0.186 | -0.132 | 0.342 | -0.418 | 0.002 | -0.477 | 0.000 | -0.467 | 0.000 | -0.236 | 0.086 |
| DG(21:5e)+NH4 | -0.077 | 0.580 | -0.120 | 0.387 | -0.487 | 0.000 | -0.539 | 0.000 | -0.432 | 0.001 | -0.332 | 0.014 |
| DG(30:2e)+H | -0.032 | 0.818 | -0.021 | 0.882 | 0.030 | 0.828 | 0.040 | 0.775 | 0.062 | 0.657 | -0.028 | 0.838 |
| LPC(20:5)+HCOO | 0.016 | 0.911 | 0.010 | 0.940 | 0.108 | 0.438 | 0.103 | 0.459 | 0.281 | 0.039 | 0.589 | 0.000 |
| PC(37:5)+H | -0.013 | 0.928 | 0.121 | 0.382 | -0.177 | 0.199 | -0.162 | 0.242 | -0.216 | 0.117 | -0.139 | 0.314 |
| PC(17:1/18:2)+H | -0.084 | 0.546 | -0.135 | 0.332 | -0.141 | 0.310 | -0.173 | 0.212 | -0.158 | 0.253 | -0.151 | 0.277 |
| PC(36:6e)+H | -0.316 | 0.020 | 0.085 | 0.539 | -0.590 | 0.000 | -0.632 | 0.000 | -0.591 | 0.000 | -0.376 | 0.005 |
| PC(18:2e/22:5)+H | -0.263 | 0.055 | -0.102 | 0.464 | -0.467 | 0.000 | -0.504 | 0.000 | -0.454 | 0.001 | -0.327 | 0.016 |
| PC(37:4e)+H | 0.084 | 0.547 | -0.033 | 0.813 | 0.303 | 0.026 | 0.300 | 0.027 | 0.269 | 0.049 | 0.394 | 0.003 |
| PC(12:0e/10:1)+H | 0.045 | 0.744 | 0.089 | 0.523 | 0.327 | 0.016 | 0.286 | 0.036 | 0.246 | 0.073 | 0.344 | 0.011 |
| PC(8:0e/6:0)+Na | 0.224 | 0.104 | 0.057 | 0.683 | 0.638 | 0.000 | 0.618 | 0.000 | 0.579 | 0.000 | 0.402 | 0.003 |
| PE(18:0/16:0)-H | 0.019 | 0.890 | -0.059 | 0.670 | 0.234 | 0.089 | 0.297 | 0.029 | 0.279 | 0.041 | 0.116 | 0.404 |
| PG(36:0/8:0)+H | -0.110 | 0.430 | -0.151 | 0.275 | 0.216 | 0.117 | 0.233 | 0.090 | 0.193 | 0.163 | 0.136 | 0.327 |
| PI(16:1)+H | -0.186 | 0.177 | -0.113 | 0.416 | -0.341 | 0.012 | -0.364 | 0.007 | -0.409 | 0.002 | -0.323 | 0.017 |
| PI(16:0/16:0)-H | 0.130 | 0.351 | 0.055 | 0.692 | 0.288 | 0.035 | 0.292 | 0.032 | 0.358 | 0.008 | 0.072 | 0.607 |
| PI(18:0/18:3)-H | 0.146 | 0.293 | -0.006 | 0.967 | 0.275 | 0.044 | 0.326 | 0.016 | 0.447 | 0.001 | 0.237 | 0.084 |
| PI(16:0/16:1)-H | 0.189 | 0.171 | 0.032 | 0.817 | 0.293 | 0.032 | 0.354 | 0.009 | 0.450 | 0.001 | 0.097 | 0.484 |
| PS(42:9e)-H | -0.159 | 0.252 | -0.096 | 0.489 | -0.526 | 0.000 | -0.566 | 0.000 | -0.504 | 0.000 | -0.287 | 0.035 |
| PS(16:1e/22:4)-H | -0.290 | 0.034 | -0.012 | 0.933 | -0.578 | 0.000 | -0.615 | 0.000 | -0.538 | 0.000 | -0.363 | 0.007 |
| SM(d43:1)+H | -0.230 | 0.094 | -0.183 | 0.184 | -0.450 | 0.001 | -0.505 | 0.000 | -0.449 | 0.001 | -0.450 | 0.001 |
| TG(20:5/18:2/20:4)+NH4 | 0.163 | 0.238 | 0.118 | 0.395 | 0.213 | 0.123 | 0.196 | 0.156 | 0.402 | 0.003 | 0.598 | 0.000 |
| TG(15:0/16:1/18:2)+NH4 | 0.031 | 0.822 | 0.036 | 0.795 | 0.100 | 0.472 | 0.119 | 0.391 | 0.174 | 0.209 | -0.010 | 0.943 |
| TG(18:0/16:0/22:1)+NH4 | 0.207 | 0.134 | -0.162 | 0.243 | 0.383 | 0.004 | 0.407 | 0.002 | 0.145 | 0.295 | 0.044 | 0.754 |
| TG(16:0/14:0/22:6)+NH4 | -0.025 | 0.856 | -0.005 | 0.973 | 0.020 | 0.884 | 0.023 | 0.870 | 0.073 | 0.598 | -0.029 | 0.837 |
| TG(16:0/20:4/20:5)+NH4 | -0.044 | 0.754 | 0.032 | 0.821 | 0.101 | 0.467 | 0.103 | 0.457 | 0.203 | 0.140 | 0.323 | 0.017 |
| TG(20:0/18:1/18:1)+NH4 | 0.231 | 0.093 | -0.086 | 0.537 | 0.472 | 0.000 | 0.484 | 0.000 | 0.354 | 0.009 | 0.095 | 0.494 |
| TG(12:0/18:2/20:4)+NH4 | 0.245 | 0.074 | 0.034 | 0.810 | 0.328 | 0.016 | 0.314 | 0.021 | 0.276 | 0.043 | 0.038 | 0.784 |
| TG(16:0/18:2/18:2)+NH4 | -0.020 | 0.885 | 0.021 | 0.879 | 0.066 | 0.635 | 0.090 | 0.518 | 0.127 | 0.360 | -0.075 | 0.592 |
| TG(16:0/14:0/18:2)+NH4 | -0.013 | 0.925 | -0.002 | 0.986 | 0.049 | 0.724 | 0.061 | 0.661 | 0.085 | 0.540 | -0.021 | 0.878 |
| TG(16:0/10:1/18:1)+NH4 | -0.075 | 0.589 | 0.104 | 0.453 | 0.031 | 0.823 | 0.051 | 0.712 | 0.056 | 0.689 | -0.014 | 0.919 |
| TG(22:4/17:1/18:2)+NH4 | 0.060 | 0.668 | -0.005 | 0.974 | 0.214 | 0.121 | 0.216 | 0.117 | 0.289 | 0.034 | 0.071 | 0.611 |
| TG(15:0/14:0/18:1)+NH4 | 0.080 | 0.564 | 0.143 | 0.301 | 0.153 | 0.270 | 0.186 | 0.178 | 0.230 | 0.095 | -0.054 | 0.697 |
| TG(18:4/18:1/18:3)+NH4 | 0.059 | 0.673 | -0.015 | 0.913 | 0.115 | 0.409 | 0.124 | 0.371 | 0.185 | 0.179 | 0.052 | 0.708 |
| TG(16:0/14:0/18:1)+NH4 | 0.055 | 0.694 | 0.082 | 0.557 | 0.157 | 0.255 | 0.179 | 0.196 | 0.199 | 0.149 | -0.035 | 0.801 |
| TG(16:0/14:1/20:5)+NH4 | -0.120 | 0.386 | -0.029 | 0.835 | -0.007 | 0.960 | 0.007 | 0.962 | 0.013 | 0.924 | 0.007 | 0.962 |
| TG(16:0/18:1/20:4)+NH4 | 0.065 | 0.642 | 0.007 | 0.957 | 0.145 | 0.294 | 0.153 | 0.269 | 0.203 | 0.141 | -0.025 | 0.858 |
| TG(14:0/18:2/20:5)+NH4 | 0.005 | 0.970 | 0.007 | 0.959 | 0.112 | 0.421 | 0.125 | 0.367 | 0.133 | 0.338 | 0.089 | 0.521 |
| TG(18:3/18:2/20:4)+Na | 0.029 | 0.837 | -0.021 | 0.881 | 0.122 | 0.380 | 0.120 | 0.388 | 0.207 | 0.132 | 0.471 | 0.000 |
| TG(15:0/14:0/18:2)+NH4 | 0.088 | 0.528 | 0.178 | 0.198 | 0.153 | 0.271 | 0.171 | 0.218 | 0.223 | 0.106 | -0.045 | 0.748 |
| TG(16:0/12:0/20:5)+Na | 0.030 | 0.828 | 0.085 | 0.541 | 0.132 | 0.340 | 0.162 | 0.240 | 0.171 | 0.216 | -0.043 | 0.758 |
| TG(18:3/18:2/20:5)+NH4 | 0.022 | 0.877 | 0.096 | 0.492 | 0.063 | 0.649 | 0.060 | 0.667 | 0.225 | 0.102 | 0.621 | 0.000 |
| TG(15:0/16:0/18:2)+NH4 | 0.150 | 0.280 | 0.148 | 0.285 | 0.201 | 0.145 | 0.217 | 0.115 | 0.273 | 0.046 | -0.041 | 0.767 |
| TG(15:0/16:0/18:1)+NH4 | 0.095 | 0.492 | 0.046 | 0.740 | 0.203 | 0.141 | 0.243 | 0.076 | 0.260 | 0.058 | -0.029 | 0.835 |
| TG(18:4/20:4/20:5)+H | 0.228 | 0.097 | -0.116 | 0.403 | 0.269 | 0.049 | 0.273 | 0.046 | 0.293 | 0.031 | 0.258 | 0.060 |
| TG(16:1/20:5/22:6)+Na | 0.018 | 0.898 | 0.098 | 0.479 | 0.017 | 0.904 | 0.017 | 0.903 | 0.268 | 0.050 | 0.678 | 0.000 |
| TG(18:1/18:2/22:4)+NH4 | 0.108 | 0.438 | -0.074 | 0.594 | 0.150 | 0.279 | 0.140 | 0.312 | 0.392 | 0.003 | 0.376 | 0.005 |
| TG(16:0/20:4/22:5)+NH4 | 0.075 | 0.589 | -0.039 | 0.780 | 0.174 | 0.208 | 0.202 | 0.144 | 0.380 | 0.005 | 0.474 | 0.000 |
| TG(16:0/16:1/22:6)+NH4 | 0.032 | 0.819 | -0.048 | 0.730 | 0.129 | 0.353 | 0.131 | 0.346 | 0.179 | 0.195 | 0.049 | 0.727 |
| TG(16:0/16:0/20:5)+NH4 | 0.040 | 0.777 | -0.007 | 0.961 | 0.075 | 0.590 | 0.085 | 0.539 | 0.121 | 0.384 | -0.023 | 0.868 |
| TG(16:0/14:0/20:5)+NH4 | -0.017 | 0.905 | -0.031 | 0.822 | 0.020 | 0.883 | 0.038 | 0.784 | 0.073 | 0.599 | -0.018 | 0.899 |
| TG(18:4/16:0/16:0)+NH4 | 0.048 | 0.728 | 0.001 | 0.994 | 0.139 | 0.317 | 0.150 | 0.278 | 0.166 | 0.231 | -0.031 | 0.825 |
| TG(12:0/18:2/18:2)+NH4 | 0.125 | 0.368 | 0.127 | 0.360 | 0.247 | 0.071 | 0.269 | 0.049 | 0.280 | 0.040 | -0.008 | 0.954 |
| TG(18:0/8:0/20:4)+Na | 0.006 | 0.968 | -0.006 | 0.967 | 0.093 | 0.504 | 0.114 | 0.412 | 0.154 | 0.267 | 0.019 | 0.889 |
