# Supplementary material for: Alterations in the Plasma Lipidome of Adult Women With Bipolar Disorder: A Mass Spectrometry-Based Lipidomics Research
Source: Front Psychiatry. 2022 Mar 21;13:802710. doi: 10.3389/fpsyt.2022.802710 (PMC8978803; doi:10.3389/fpsyt.2022.802710)
Supplement: Supplementary Table 5 — ROC analysis for altered lipids at species level. [file Table_5.doc]

Supplemental table 5. ROC analysis for altered lipids at species level

| Species | AUC | 95% CI | *P* value |
| --- | --- | --- | --- |
| PS(42:9e)-H | 1 | 1~1 | 4.70E-09 |
| DG(21:5e)+NH4 | 0.9972 | 0.9906~1 | 1.81E-08 |
| PC(36:6e)+H | 0.9972 | 0.9906~1 | 5.55E-14 |
| PC(8:0e/6:0)+Na | 0.9861 | 0.9642~1 | 3.20E-10 |
| PS(16:1e/22:4)-H | 0.9583 | 0.9017~1 | 4.01E-12 |
| TG(16:0/16:1/22:6)+NH4 | 0.9417 | 0.8784~1 | 0.007759 |
| TG(16:0/20:4/22:5)+NH4 | 0.9222 | 0.8525~0.9919 | 1.37E-05 |
| TG(22:4/17:1/18:2)+NH4 | 0.9111 | 0.8323~0.9899 | 0.001528 |
| TG(18:0/8:0/20:4)+Na | 0.9111 | 0.8291~0.9931 | 0.017032 |
| TG(18:1/18:2/22:4)+NH4 | 0.8917 | 0.8072~0.9762 | 0.000308 |
| AcCa(14:2)+H | 0.8861 | 0.8005~0.9717 | 1.85E-07 |
| PC(18:2e/22:5)+H | 0.8833 | 0.7964~0.9703 | 1.17E-07 |
| LPC(20:5)+HCOO | 0.875 | 0.7777~0.9723 | 0.002894 |
| SM(d43:1)+H | 0.8722 | 0.7689~0.9756 | 7.38E-07 |
| PI(16:1)+H | 0.8694 | 0.7758~0.963 | 6.60E-06 |
| PE(18:0/16:0)-H | 0.8556 | 0.7545~0.9566 | 0.000279 |
| DG(20:4e)+NH4 | 0.8389 | 0.7323~0.9455 | 1.76E-06 |
| PC(12:0e/10:1)+H | 0.8389 | 0.719~0.9588 | 0.004834 |
| TG(18:4/20:4/20:5)+H | 0.8306 | 0.7255~0.9356 | 0.000426 |
| AcCa(14:1)+H | 0.825 | 0.7161~0.9339 | 4.71E-06 |
| TG(12:0/18:2/18:2)+NH4 | 0.8167 | 0.6909~0.9424 | 0.013841 |
| PI(18:0/18:3)-H | 0.8139 | 0.688~0.9398 | 0.000237 |
| TG(16:0/14:1/20:5)+NH4 | 0.8111 | 0.697~0.9252 | 0.088495 |
| TG(16:0/20:4/20:5)+NH4 | 0.8056 | 0.6884~0.9227 | 0.002775 |
| TG(20:0/18:1/18:1)+NH4 | 0.8028 | 0.662~0.9436 | 0.00023 |
| TG(15:0/16:0/18:1)+NH4 | 0.8 | 0.6747~0.9253 | 0.010915 |
| PI(16:0/16:0)-H | 0.7944 | 0.6725~0.9164 | 0.003485 |
| PI(16:0/16:1)-H | 0.7889 | 0.6621~0.9157 | 0.001028 |
| TG(18:4/16:0/16:0)+NH4 | 0.775 | 0.6516~0.8984 | 0.016434 |
| TG(16:0/16:0/20:5)+NH4 | 0.7611 | 0.6319~0.8904 | 0.036212 |
| TG(18:3/18:2/20:4)+Na | 0.75 | 0.6202~0.8798 | 0.001936 |
| TG(16:0/12:0/20:5)+Na | 0.7417 | 0.6077~0.8757 | 0.029951 |
| TG(16:0/18:1/20:4)+NH4 | 0.7389 | 0.6038~0.874 | 0.014568 |
| TG(12:0/18:2/20:4)+NH4 | 0.7361 | 0.5974~0.8749 | 0.005622 |
| TG(16:0/10:1/18:1)+NH4 | 0.725 | 0.5875~0.8625 | 0.150323 |
| PG(36:0/8:0)+H | 0.7083 | 0.5574~0.8593 | 0.014432 |
| TG(16:1/20:5/22:6)+Na | 0.7083 | 0.5603~0.8563 | 0.021681 |
| TG(18:3/18:2/20:5)+NH4 | 0.7028 | 0.5625~0.8431 | 0.029829 |
| TG(18:0/16:0/22:1)+NH4 | 0.6889 | 0.5307~0.8471 | 0.005928 |
| TG(16:0/14:0/18:1)+NH4 | 0.6806 | 0.5337~0.8274 | 0.020943 |
| TG(14:0/18:2/20:5)+NH4 | 0.6806 | 0.5307~0.8304 | 0.009956 |
| TG(18:4/18:1/18:3)+NH4 | 0.6778 | 0.5222~0.8333 | 0.01614 |
| TG(20:5/18:2/20:4)+NH4 | 0.6611 | 0.4955~0.8267 | 0.000525 |
| TG(16:0/14:0/18:2)+NH4 | 0.6583 | 0.5058~0.8108 | 0.055628 |
| TG(15:0/16:1/18:2)+NH4 | 0.65 | 0.4982~0.8018 | 0.045774 |
| TG(15:0/14:0/18:2)+NH4 | 0.65 | 0.4889~0.8111 | 0.104104 |
| TG(15:0/16:0/18:2)+NH4 | 0.6444 | 0.4947~0.7942 | 0.054925 |
| DG(30:2e)+H | 0.625 | 0.4719~0.7781 | 0.066562 |
| TG(15:0/14:0/18:1)+NH4 | 0.6194 | 0.4647~0.7741 | 0.062979 |
| PC(37:4e)+H | 0.6139 | 0.4564~0.7714 | 0.012251 |
| TG(16:0/14:0/20:5)+NH4 | 0.5917 | 0.4327~0.7506 | 0.055857 |
| PC(37:5)+H | 0.5028 | 0.3381~0.6674 | 0.023028 |
| PC(17:1/18:2)+H | 0.4556 | 0.2966~0.6145 | 0.028812 |
| TG(16:0/18:2/18:2)+NH4 | 0.4556 | 0.2905~0.6206 | 0.064922 |
| TG(16:0/14:0/22:6)+NH4 | 0.4417 | 0.2824~0.6009 | 0.103241 |

ROC: operating characteristic; AUC: the area under the ROC curve; CI: confidence interval.
